# Altered secretory and neuroprotective function of the choroid plexus in progressive multiple sclerosis

**DOI:** 10.1186/s40478-020-00903-y

**Published:** 2020-03-19

**Authors:** Sabela Rodríguez-Lorenzo, David Miguel Ferreira Francisco, Ricardo Vos, Bert van het Hof, Merel Rijnsburger, Horst Schroten, Hiroshi Ishikawa, Wissam Beaino, Rémy Bruggmann, Gijs Kooij, Helga E. de Vries

**Affiliations:** 1 Department of Molecular Cell Biology and Immunology, Amsterdam Neuroscience, MS Center Amsterdam, Amsterdam UMC, Vrije Universiteit Amsterdam, de Boelelaan 1117, 1007 MB Amsterdam, Netherlands; 2 Interfaculty Bioinformatics Unit and Swiss Institute of Bioinformatics, University of Bern, Bern, Switzerland; 3 Department of Radiology & Nuclear Medicine, Amsterdam UMC, Amsterdam, the Netherlands; 4 Pediatric Infectious Diseases, University Children’s Hospital Manheim, Medical Faculty Manheim, Heidelberg University, Manheim, Germany; 5 Laboratory of Clinical Regenerative Medicine, Department of Neurosurgery, Faculty of Medicine, University of Tsukuba, Tsukuba, Japan; 6 Medical Biochemistry, Amsterdam Cardiovascular Sciences, Amsterdam UMC, University of Amsterdam, Meibergdreef 9, Amsterdam, 1105 AZ the Netherlands

**Keywords:** Choroid plexus, Multiple sclerosis (MS), RNA-sequencing, Cerebrospinal fluid (CSF), Hypoxia, PAI-1

## Abstract

The choroid plexus (CP) is a key regulator of the central nervous system (CNS) homeostasis through its secretory, immunological and barrier properties. Accumulating evidence suggests that the CP plays a pivotal role in the pathogenesis of multiple sclerosis (MS), but the underlying mechanisms remain largely elusive. To get a comprehensive view on the role of the CP in MS, we studied transcriptomic alterations of the human CP in progressive MS and non-neurological disease controls using RNA sequencing. We identified 17 genes with significantly higher expression in progressive MS patients relative to that in controls. Among them is the newly described long non-coding RNA *HIF1A-AS3*. Next to that, we uncovered disease-affected pathways related to hypoxia, secretion and neuroprotection, while only subtle immunological and no barrier alterations were observed. In an ex vivo CP explant model, a subset of the upregulated genes responded in a similar way to hypoxic conditions. Our results suggest a deregulation of the Hypoxia-Inducible Factor (HIF)-1 pathway in progressive MS CP. Importantly, cerebrospinal fluid levels of the hypoxia-responsive secreted peptide PAI-1 were higher in MS patients with high disability relative to those with low disability. These findings provide for the first time a complete overview of the CP transcriptome in health and disease, and suggest that the CP environment becomes hypoxic in progressive MS patients, highlighting the altered secretory and neuroprotective properties of the CP under neuropathological conditions. Together, these findings provide novel insights to target the CP and promote the secretion of neuroprotective factors into the CNS of progressive MS patients.

## Introduction

Multiple sclerosis (MS) is a chronic demyelinating disorder of the central nervous system (CNS) affecting 2.3 million people worldwide [59]. Most MS patients initially present a relapsing-remitting phase, in which peaks of the disease coincide with the appearance or reactivation of inflammatory lesions in the brain and spinal cord [46], followed by periods of inactivity. Eventually, irreversible neurological damage accumulates, and secondary progressive MS develops. A small subset of patients follows a progressive course from the onset of the disease, known as primary progressive MS. Neurodegeneration, brain atrophy and a steady increase of clinical disability are features of both primary and secondary progressive MS, together affecting 43% of MS patients [7]. While several treatments are available for the inflammation-driven relapsing-remitting phase of MS, immune-modulators are less effective in progressive MS, indicating a need for new restorative therapies for this disease phase. Inflammation is present in all stages of MS, but in progressive MS it may be trapped behind the restored blood brain barrier, making it thus inaccessible to immunomodulatory treatments [39]. Moreover, oxidative stress and iron accumulation can magnify neurological damage in progressive MS [39]. Thus, the development of novel therapies that halt the neurodegenerative process and/or promote neuroprotection may be vital to combat progressive MS.

The choroid plexus (CP) is a strategically located villous structure that actively regulates CNS homeostasis [5]. There are four CP located in each of the brain ventricles, consisting of highly vascularized stroma surrounded by a tight layer of epithelial cells that form the blood-cerebrospinal fluid barrier (BCSFB) [22]. The CP is the main producer of cerebrospinal fluid (CSF), and secretes a remarkable amount of signaling and trophic factors into the CNS. Moreover, the CP is capable of sensing and integrating stimuli from the periphery and CNS and coordinate secretory responses [22].

In the past years, there is increasing evidence of the involvement of the CP in a variety of disorders such as ageing [3, 16, 83], Alzheimer’s disease [30, 70], frontotemporal dementia [70], schizophrenia [34], Huntington’s disease [70] and cerebral ischemia [66]. In MS, the presence of periventricular lesions and the increased numbers of immune cells in the CSF [11] suggest an involvement of the CP in regulating pathology. In the mouse model for MS, called experimental autoimmune encephalomyelitis (EAE), the first wave of immune cell infiltration is thought to occur through the CP, by means of the interaction of Th17 cells with the CP epithelium-derived chemokine CCL20 [58]. In humans, activation of immune cell populations and adhesion mechanisms have been described in the CP from MS patients [65, 77]. Alterations in the BCSFB that could result in immune cell infiltration into the CNS have also been studied both in EAE and MS, with variable results [13, 37]. However, changes in the secretory activity of the CP in MS have not been explored. Given its critical contribution to CNS homeostasis through the production of CSF, the CP is a strong candidate to influence MS pathogenesis.

To get a comprehensive overview of CP alterations in progressive MS and reveal potential disruptions in any of its critical attributes, including secretion, barrier formation or the immunological milieu, we performed RNA sequencing (RNA-seq) of human postmortem CP samples from the lateral ventricle of progressive MS patients and non-neurological controls. We here provide an overview on the CP transcriptomic alterations in progressive MS, including hypoxia-responsive, neuroprotective and secretory changes. Our findings suggest a deregulation of the Hypoxia-Inducible Factor (HIF)-1 pathway in progressive MS CP. These transcriptional changes are accompanied by altered levels of the hypoxia-responsive secreted peptide plasminogen activator inhibitor-1 (PAI-1) in the CSF of MS patients. Our data suggest that the CP epithelium is a source of secreted peptides into the CSF during progressive MS that can regulate CNS homeostatic responses. Together, this study provides the first characterization of the transcriptomic profile of the CP in progressive MS, pointing to hypoxia-related and neuroprotective responses and to a dysregulation in peptide secretion which may have important effects on CNS homeostasis during MS pathogenesis.

## Materials and methods

### Human samples

Tissue was obtained from patients with clinically diagnosed and neuro-pathologically confirmed progressive MS as well as from control cases without neurological disease by rapid autopsy and immediately frozen in liquid nitrogen. CP tissue from the lateral ventricles was obtained from the Netherlands Brain Bank, while brain tissue was obtained from the UK MS Society Tissue Bank, Imperial College London. All parties received permission to perform autopsies, for the use of tissue and for access to medical records for research purposes. All donors, or their next of kin, had given informed consent for autopsy and use of their brain tissue for research purposes. Relevant clinical information of CP donors for RNA-seq is summarized in Supplementary Table 1. For CP culturing experiments, donor information is summarized in Supplementary Table 2. Clinical data of brain donors are listed in Supplementary Table 4. Lesions were immunohistochemically characterized as areas with abundant immune cell infiltrates (MHCII+ cells) and extensive myelin loss (proteolipid protein, PLP).

CSF samples were obtained from the Karolinska Institute biobank containing samples collected during routine neurological diagnostic work-up. A total of 58 subjects were included in this study. MS patients fulfilling the McDonald criteria included RRMS, SPMS and PPMS. The control cohort included non-inflammatory or inflammatory neurological disease controls, symptomatic controls, and healthy controls. Classification and scoring of MS patients was assessed by trained neurologists. For RRMS, a relapse was defined as an increase with ≥1 point on the EDSS with a duration of at least 1 week before sampling, while remission was defined as a stable status longer than 3 months prior to sampling. SPMS was defined as an initial relapsing-remitting disease course followed by more than 12 months of continuous worsening of neurological function, with or without occasional relapses. At time of sampling, none of the patients were treated with immunomodulatory drugs. CSF donors received verbal and written information and gave consent in writing before inclusion in the study. CSF samples were centrifuged immediately after sampling to isolate the cells and larger particles and stored frozen at − 80 °C until analysis. CSF sample information is summarized in Supplementary Table 3.

### RNA sequencing and analysis

Up to 30 mg of tissue was collected from the whole lateral CP by cutting slices approximately 10 μm thick using a cyrotome at -20 °C. Tissue was homogenized with a POLYTRON PT 1200 (Kinematica). Total RNA from the CP was extracted with the RNeasy mini kit (Qiagen, 74104), following the manufacturer’s instructions. After extraction, the quality of the RNA was assessed using a Fragment Analyzer. Samples with RIN values ≥ 6.5 were selected (Supplementary Table 1). Of these, 6 were from non-neurological controls (4 females, 2 males; average age of 62 ± 6.11) and the remaining 6 from individuals with diagnosed progressive MS (2 females, 4 males; average age of 61 ± 11.15) (Supplementary Table 1). The RNA libraries for the selected 12 samples were prepared according to the manufacturer’s instructions using TruSeq stranded mRNA Library Prep Kit including an rRNA depletion step (RiboMinus, ThermoFisher). Sequencing was performed at the Next Generation Sequencing (NGS) Platform of the University of Bern using a HiSeq3000 producing paired-end reads with an average depth of approximately 30 million reads per sample (ranging approximately from 28 to 32 million reads) with a read length of 2 × 150 bases.

RNA-seq analysis was performed as follows: the quality of the fastq files was assessed using FASTQC software [4] (version 0.11.5). The reads were aligned to the human reference genome (version GRCh38) using TopHat2 [33] (version 2.1.1). Qualimap [21] (version 2.2.1) was used for quality control and IGV [63] (version 2.3.69) for visualization of the aligned reads. Read counts by transcript were determined by using HTSeq-count [32] (version 0.6.1). Normalization and differential expression analysis were performed with the DESeq2 package [45] (Version 1.16.1) in R [74] (Version 3.4.0). The SVA (Version 3.24.4) package [41] was used to test for batch effects among the samples. Genes with an adjusted *p*-value (False Discovery Rate) ≤ 0.05 were selected for further analysis.

Differential alternative splicing analysis was performed based on the same fastq files as for the gene level based RNA-Seq analysis. The reads were aligned to the human reference genome (version GRCh38) using Hisat2 [32] (Version 2.1.0) and subsequently sorted by coordinates using Samtools [42] (version 1.3). Stringtie [57] (version 1.3.3b) was then used to predict, merge and estimate the abundance of the different transcripts. Finally, differential expression assessment, visualization and annotation of the transcripts was done in R [74] (Version 3.4.3) using the Ballgown package [19] (Version 2.10.0) with parallel use of the Limma [61] (Version 3.34.0) and edgeR [64] packages (version 3.20.1) for analysis with regularization.

### Validation of differentially expressed genes by qPCR

To validate differentially expressed genes from the RNA-seq experiment, the same RNA of CP samples was analyzed by quantitative PCR (qPCR). RNA was reverse-transcribed into cDNA with the High-Capacity cDNA Reverse Transcription Kit (4368814, Thermo Fisher Scientific). qPCR reactions were performed in technical duplicates on an Applied Biosystems Viia7 real-time PCR machine (Thermo Fisher Scientific) using SYBR green detection. Primers were obtained from Thermo Fisher Scientific, and their sequences are given in Supplementary Table 5. Normfinder was used to select *GAPDH* and *18 s rRNA* as the most stable reference genes in MS and control CP among a set of 8 candidates.

### Choroid plexus explants culture

Fresh human postmortem CP was stored in Hibernate A medium (A1247501, Thermo Fisher Scientific) for less than 24 h. The CP was washed twice in cold PBS and cut with sharp scissors into small pieces (around 3 mm^2^ each). Pieces from one donor were split into two different plates with DMEM/F12 (31330–038, Gibco) supplemented with 10% (v/v) heat inactivated FCS, and 1% PSG). Both plates with explants were cultured under normoxic conditions (20% O_2_) for 24 h, after which one plate was switched to hypoxia (1% O_2_) for another 24 h. Then, CP explants were washed twice with cold PBS and RNA extraction was performed with TrizOL (15596026, Invitrogen) using a blend homogenizer and a 21G syringe for tissue disruption.

### Immunoassays of ADM, PAI-1 and STC2 in CSF

Determination of ADM protein concentration in human CSF was performed with a competitive radioimmunoassay method (RK-010-01, Phoenix Pharmaceuticals), following the manufacturer’s instructions. CSF samples were concentrated 5X by lyophilization and resolubilization in sterile water to adjust to the detection range of the assay (100–12,800 pg/mL). PAI-1 concentration in human CSF was measured using a sandwich ELISA (ab108891, Abcam) following the manufacturer’s instructions. STC2 concentration in human CSF samples was measured using a sandwich ELISA (AL-143, AnshLabs) following the manufacturer’s instructions. Investigators were blinded to sample type during experiments and data collection.

### RNA isolation from brain MS lesions and control tissue

Active and chronic active MS lesions and white matter from controls were dissected from frozen brain blocks. To this aim, lesions were first outlined according to their myelin (PLP) and inflammatory (MHCII) status, using a sharp needle. Thereafter, 10 μm sections were cut, and lesion area and normal appearing white matter were collected separately in tubes and kept in liquid nitrogen. Messenger RNA isolation was conducted using the Qiagen RNeasy Lipid Tissue Mini Kit (Qiagen, the Netherlands) according to manufacturer’s instructions.

### Choroid plexus epithelial cell culture

HIBCPP cells (from human choroid plexus papilloma cell line [29]) were cultured in DMEM/F12 (31330–038, Gibco) supplemented with 4.8 mM L-Glutamine (25030–024, Gibco), 5 μg/mL insulin (I9278, Sigma), penicillin (100 U/ml) and streptomycin (100 μg/ml), 15% (v/v) heat inactivated fetal calf serum (FCS). For hypoxia experiments, cells were cultured under normoxic (20% O_2_) or hypoxic (1% O_2_) conditions for 24 h. For inflammation experiments, cells were treated with 10 ng/mL recombinant human TNF-ɑ (300-01A) for 24 h. Then, cells were washed twice with cold PBS and RNA extraction was performed with TrizOL (15596026, Invitrogen).

### Gene expression analysis of HIBCPP cells, CP explants and brain tissue: RT-qPCR

cDNA synthesis was performed using a High-Capacity cDNA Reverse Transcription Kit (4368814, Thermo Fisher Scientific). qPCR reactions were performed in technical duplicate on a Viia7 real-time PCR machine (Thermo Fisher Scientific) using SYBR green detection. Primers were obtained from Thermo Fisher Scientific, and their sequences are given in Supplementary Table 5. Messenger RNA expression levels were normalized using *18 s rRNA* or *GAPDH*.

### Statistical analysis

Statistical analysis was done using R statistical software version 3.4.0 (2017-04-21). Differences in qPCR gene expression results were tested by two-tailed Welch t-test, except for CP explant data, where differences were tested by paired two-tailed Welch t-test. Immunoassay protein concentration results are expressed as median and interquartile ranges (IQR). The Shapiro-Wilk’s test was used to assess the normality of distribution of investigated parameters*.* The F test was used to assess heteroscedasticity. Outliers were investigated by Grubbs’, Dixon and Chi-squared tests. Differences in normally distributed data were tested by two-tailed Welch t-test, while a two-tailed Wilcoxon rank sum test (Mann–Whitney U test) with continuity correction was used for non-normally distributed data. Pearson’s correlation was used to analyze the association between studied parameters. For dichotomization of the cohort by EDSS score, a cut-off of 5.5 was chosen, based on the EDSS median in the cohort and the clinical significance of the score [51].

## Results

### Transcriptional alterations in the CP of progressive MS patients

**Fig. 1:**
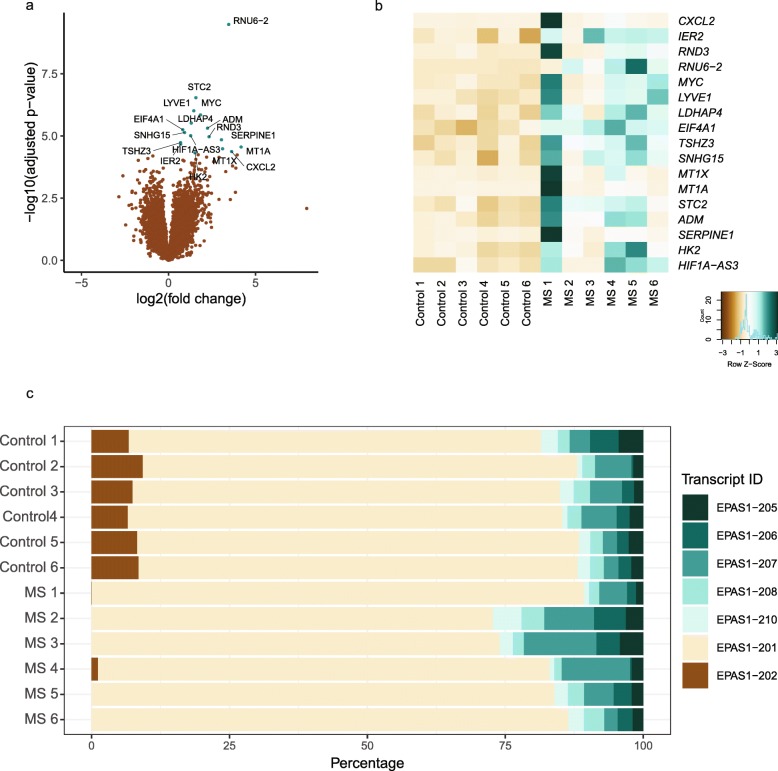
Transcriptional profile of the human choroid plexus in progressive MS. **a** Volcano plot of statistical significance against fold change between CP of progressive MS cases (*n* = 6) and that of controls (n = 6). Each dot represents a gene. Genes considered to be significantly differentially expressed (adjusted p-value < 0.05) are depicted in turquoise. **b** Heatmap of differentially expressed genes (adjusted p-value < 0.05) between progressive MS and control CP. Samples are ordered by sample name. **c** 100% stacked chart for *EPAS1* displaying the percentages of each splice variant in progressive MS and control CP. Samples are ordered by sample name. Splice variant IDs are the versions from Ensembl Human GRCh38 build 97 as of submission

To reveal potential transcriptomic changes associated with progressive MS, we performed RNA-seq of 6 human CP samples from progressive MS patients and 6 non-neurological disease control CP samples. Differential expression analysis revealed a total of 17 genes with higher expression in the CP of progressive MS patients compared with that of non-neurological controls (Table 1, Fig. 1a and b), whereas we did not observe genes with lower expression. Several of the identified genes are involved in hypoxia, such as *ADM*, *HK2, STC2, SERPINE1*, *SNHG15* and possibly *HIF1A-AS3*, but also in neuroprotection, such as *ADM*, *SERPINE1, MT1A* and *STC2*. These transcriptional alterations were validated in the same human CP samples by qPCR (Supplementary 1a). Accordingly, Gene Ontology (GO) term enrichment analysis in the dataset showed overrepresentation of genes related to hypoxia (‘response to hypoxia’, GO:0001666, *p* = 0.001; ‘cellular response to hypoxia’ GO:0071456, *p* = 0.007) (Supplementary Table 6). Surprisingly, only one of the differentially expressed genes, *CXCL2*, appeared to be directly linked to inflammation, although there was an enrichment in several GO terms related to inflammatory processes (Supplementary Table 6).

**Table 1:** List of differentially expressed genes between MS and control CP

Description	Gene symbol	Ensembl ID	Mean Normalized Reads	log2FoldChange	Adjusted *p*-value
RNA U6 small nuclear 2	RNU6–2	ENSG00000207357	19.74	3.46	5.86E-06
stanniocalcin 2	STC2	ENSG00000113739	81.46	1.57	0.003
lymphatic vessel endothelial hyaluronan receptor 1	LYVE1	ENSG00000133800	3253.05	1.45	0.006
MYC proto-oncogene, bHLH transcription factor	MYC	ENSG00000136997	236.37	1.84	0.006
lactate dehydrogenase A pseudogene 4	LDHAP4	ENSG00000214110	122.50	1.31	0.011
adrenomedullin	ADM	ENSG00000148926	1046.05	2.24	0.014
eukaryotic translation initiation factor 4A1	EIF4A1	ENSG00000161960	48.80	0.82	0.014
small nucleolar RNA host gene 15	SNHG15	ENSG00000232956	94.54	0.92	0.016
HIF1A antisense RNA 3	HIF1A-AS3	ENSG00000258667	63.96	1.27	0.019
Rho family GTPase 3	RND3	ENSG00000115963	451.98	2.33	0.019
serpin family E member 1	SERPINE1	ENSG00000106366	930.69	3.04	0.023
teashirt zinc finger homeobox 3	TSHZ3	ENSG00000121297	109.95	0.69	0.027
immediate early response 2	IER2	ENSG00000160888	231.88	0.70	0.029
metallothionein 1A	MT1A	ENSG00000205362	71.35	4.17	0.035
metallothionein 1X	MT1X	ENSG00000187193	34.07	3.11	0.039
C-X-C motif chemokine ligand 2	CXCL2	ENSG00000081041	127.95	3.63	0.047
hexokinase 2	HK2	ENSG00000159399	572.79	1.52	0.050

In addition to the RNA-seq differential gene expression analysis, we performed differential alternative splicing analysis. Among the whole genome, the expression of one of the transcripts of the gene *EPAS1* (EPAS1–202), involved in hypoxic responses [80], was either completely or nearly lost in the progressive MS samples (Fig. 1c and Supplementary 1b). Together, these results indicate that transcriptomic alterations are apparent in the CP of progressive MS patients, involving hypoxia- and neuroprotection-related genes.

### Hypoxia responses in human CP explants

**Fig. 2:**
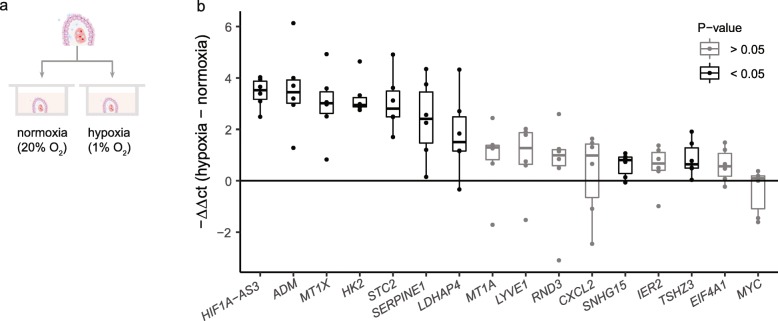
Hypoxia responses in human CP explants. **a** Schematic representation of the experimental setup of CP explants cultured in hypoxia and normoxia. **b** Difference in relative gene expression between human postmortem CP paired samples from each donor (*n* = 6) incubated 24 h in hypoxia (1% O_2_) or normoxia (20% O_2_), analyzed using RT-qPCR. Data are normalized to *18 s rRNA*. Results are displayed as the negative difference in Ct values between hypoxia and normoxia (represented as -ΔΔCt) and presented as median with confidence interval. Differences were tested by paired two-tailed Welch t-test

Our RNA-seq analysis revealed the induction of hypoxia responsive genes in the CP of progressive MS patients, suggesting a hypoxic CP environment during the disease. To validate these findings, we studied the effect of CP hypoxia on the differentially expressed hypoxia responsive genes, as assessed by RNA-seq. For this, we cultured human postmortem CP explants in hypoxic (1% O_2_) and normoxic (20% O_2_) conditions for 24 h (Fig. 2a). We detected a higher expression of known hypoxia-related genes (*HK2*, *ADM*, *STC2*, *SERPINE1* and *SNHG15*), but also of other genes, to our knowledge, previously unrelated to hypoxia (*HIF1A-AS3*; *MT1X*, *TSHZ3*, *LDHAP4*) (Fig. 2b and Supplementary Table 7). Thus, the transcriptional response to hypoxia in our CP explant model is compatible with the presence of a hypoxic environment in the CP of progressive MS patients.

### Higher PAI-1 (*SERPINE1*) concentration in the CSF of MS patients with high disability

**Fig. 3:**
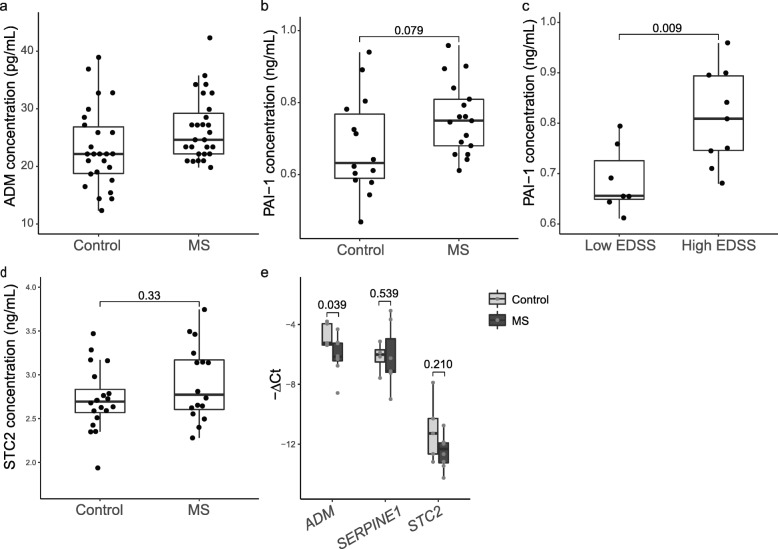
Altered concentration of ADM and PAI-1 peptides in the CSF of MS patients. **a** ADM protein concentration in CSF biopsies from MS patients (*n* = 27) and controls (*n* = 26), as measured by RIA. Differences were tested by two-tailed Wilcoxon rank sum test (Mann–Whitney U test) with continuity correction (*p* = 0.034). **b** PAI-1 protein concentration in CSF biopsies from MS patients (*n* = 17) and controls (*n* = 14), as measured by ELISA. Differences were tested by unpaired two-tailed classic t-test. **c** PAI-1 protein concentration in the CSF of MS patients with high disability (high EDSS; *n* = 9) or low disability (low EDSS; *n* = 7), as measured by ELISA. Differences were tested by unpaired two-tailed classic t-test. **d** STC2 protein concentration in CSF biopsies from MS patients (n = 16) and controls (*n* = 20), as measured by ELISA. Differences were tested by unpaired two-tailed classic t-test. In **a-d** results are displayed as median and interquartile ranges (IQR). **e***ADM*, *SERPINE1* and *STC2* mRNA expression in brain lesions of MS patients (*n* = 8) compared to white matter from controls (*n* = 5), as assessed by RT-qPCR. Data are normalised to *GAPDH*. Results are displayed as fold change relative to control white matter and presented as mean ± SEM. Differences were tested by two-tailed Welch t-test

The CP has an important secretory function which is key in the maintenance of CNS homeostasis. Our RNA-seq analysis revealed three disease-affected genes encoding for the secreted proteins adrenomedullin (ADM), plasminogen activator inhibitor-1 (PAI-1) and stanniocalcin-2 (STC2) (Fig. 1b). To investigate the alterations of the CP secretory function in MS we determined the concentration of ADM, PAI-1 and STC2 in the CSF of MS patients and controls. There was a trend of higher ADM levels in the CSF from MS patients compared to those in controls (24.61 pg/mL ± 7.05 vs 22.16 pg/mL ± 8.09; W = 470.5, *p* = 0.034) (Fig. 3a). However, a weak but significant positive correlation between ADM and age was observed (r = 0.3, *p* = 0.025) (Supplementary 3a), while MS cases were significantly older than the controls (Supplementary 3b). There was no correlation with EDSS (Supplementary 3c-d). PAI-1 concentration in the CSF of controls was 0.63 ng/mL (± 0.18 IQR) while in the CSF of MS patients the concentration was 0.75 ng/mL (± 0.13 IQR), but this difference was not significant (t = 1.82, *p* = 0.079) (Fig. 3b). However, PAI-1 levels in the CSF were positively correlated with age (r = 0.66, *p* = 5.05e-05) (Supplementary 3e). Interestingly, we did observe significantly higher levels of PAI-1 in the CSF of MS patients with more clinical disability, reflected by the EDSS score (0.81 ng/mL ± 0.15 IQR, EDSS score > 5.25) compared with MS patients with a lower disability score (0.66 ng/mL ± 0.08 IQR, EDSS score < 5.25) (t = 2.89, *p* = 0.009) (Fig. 3c) while the age between these two groups was similar (Supplementary 3f). Lastly, STC2 levels in the CSF from MS patients (2.77 ng/mL ± 0.54 IQR) did not differ from those in controls (2.69 ng/mL ± 0.27 IQR; t = − 1.317, *p* = 0.198) (Fig. 3d) or between disability states (Supplementary 3h-i), but STC2 concentration in the CSF was positively correlated with age (r = 0.409, *p* = 0.012) (Supplementary 3g).

We next hypothesized that the elevated levels of PAI-1 in the CSF of MS patients originate from the CP. However, as this protein may also be expressed in the brain parenchyma [82], we studied gene expression in white matter lesions from progressive MS patients and healthy control brain tissue. Our results show no differences in *SERPINE1* (PAI-1) or *STC2* expression between the two groups, and even lower expression levels of *ADM* (t = − 2.338, *p* = 0.039) are present in MS lesions relative to control (Fig. 3e). In addition to our data from MS lesions, we analyzed an available microarray gene expression dataset of MS brain lesions and control white matter (GEO accession ID: GSE38010) [2, 27]. Similarly to our results, we found lower *ADM* expression levels in MS lesions compared to control white matter (adjusted *p* = 0.067, log2FC = − 2.19), while *SERPINE1* and *STC2* expression did not differ (Supplementary Fig. 4). These findings suggest that the transcriptomic alterations in the CP of progressive MS patients result in the increased secretion of proteins such as PAI-1 into the CSF.

### Expression of *ADM*, *SERPINE1* (PAI-1) and *STC2* in choroid plexus epithelial cells

**Fig. 4:**
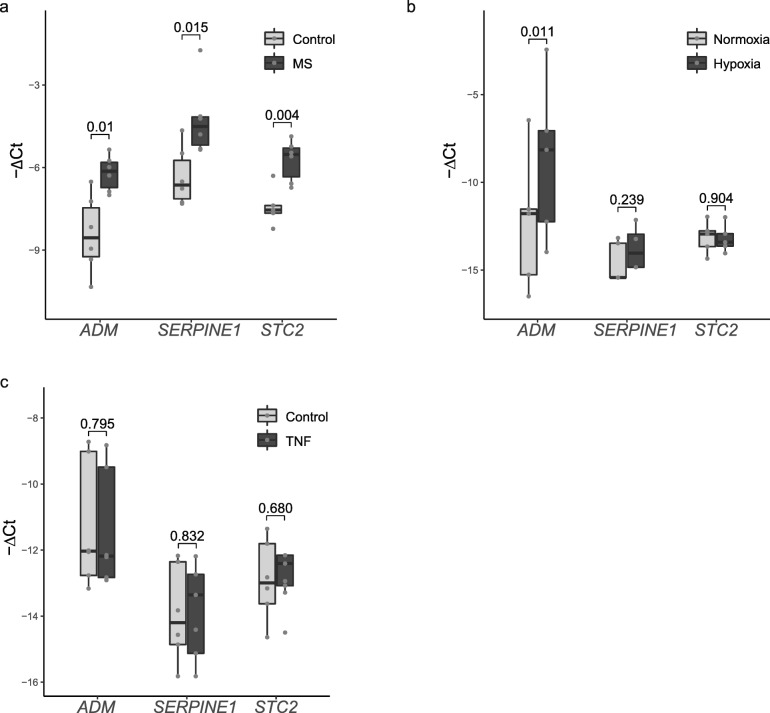
Expression of *ADM*, *SERPINE1* (PAI-1) and *STC2* by choroid plexus epithelial cells. **a***ADM*, *SERPINE1* and *STC2* mRNA expression in the CP of progressive MS patients and controls as assessed by RT-qPCR. Data are normalised to both *18 s rRNA* and *GAPDH*. Results are displayed as fold change relative to control CP (n = 6 CP per group) and presented as mean ± SEM. Differences were tested by two-tailed Welch t-test. **b***ADM, SERPINE1* and *STC2* mRNA expression in human choroid plexus epithelial cells (HIBCPP) exposed to hypoxic (1% O_2_) or normoxic (20% O_2_) conditions. Data are normalised to *18 s rRNA*. Results are displayed as the negative difference in Ct values between each gene and *18 s rRNA* (−ΔCt) and presented as median and interquartile ranges (IQR). Data from triplicate wells in 5 to 6 independent experiments. Differences were tested by two-tailed Welch t-test. **c***ADM*, *SERPINE1* and *STC2* mRNA expression in human choroid plexus epithelial cells (HIBCPP) exposed to the inflammatory cytokine TNFα (10 ng/mL). Data are normalised to *18 s rRNA*. Results are displayed as the negative difference in Ct values between each gene and *18 s rRNA* (−ΔCt) and presented as median and interquartile ranges (IQR). Data from triplicate wells in 6 independent experiments. Differences were tested by two-tailed Welch t-test

CP epithelial cells are the main producer of CSF, and ADM and PAI-1 are known to be secreted by CP epithelium [71, 72, 76, 79]. In order to confirm the epithelial origin of the enhanced expression of *ADM*, *PAI-1* and *STC2* in progressive MS (Fig. 4a), we set out to study their gene expression in human CP epithelial cells (HIBCPP cells). Expression levels of *ADM* were higher under hypoxic compared to normoxic culture conditions (t = 2.725, *p* = 0.011, Fig. 4b), while we did not see differences in the expression of *SERPINE1* or *STC2* (Fig. 4b). In contrast, CP epithelial cells exposed to an inflammatory stimulus (10 ng/mL TNF-ɑ) did not show altered expression of *ADM, SERPINE1* or *STC2* relative to control (Fig. 4c). These results indicate that the CP epithelial cells express *ADM*, *SERPINE1* and *STC2* and that ADM levels are increased upon hypoxic conditions. Together with the CP explant data (Fig. 2), our results are consistent with the presence of hypoxia in the CP of progressive MS patients.

## Discussion

In this study, we explored the transcriptional profile of the CP in progressive MS patients compared to non-neurological controls and how this relates to altered peptide concentrations in the CSF of MS patients. We found an enrichment in hypoxia-related, neuroprotective and secretory genes among those differentially expressed, uncovering new important roles of the CP in the pathogenesis of MS. Since the CP is the main producer of CSF, the relevance of its secretory function was further validated by studying the levels of three of the identified proteins, ADM, PAI-1 (*SERPINE1*) and STC2, in the CSF of MS patients. We observed a higher concentration of PAI-1 in patients with more disease severity. Finally, we provide supporting evidence for the CP epithelium as the source of these peptides.

To our knowledge, this is the first transcriptomic characterization of the progressive MS CP in a human setting. RNA-seq of the CP in other neurological diseases such as frontotemporal dementia, Alzheimer’s and Huntington’s disease [70], or schizophrenia [34] revealed alterations in the barrier or immune homeostatic properties of the CP. In progressive MS, we found predominantly alterations related to hypoxia but also secretion and neuroprotection, possibly reflecting the particular etiology of MS.

The postmortem tissue used in this study was carefully selected for high RNA integrity, thereby limiting the availability of samples. Although donors with other neurological diseases were excluded from the study, the high variability of human samples is reflected in our dataset (Supplementary 1d). Additionally, the CP consists of a variety of cells, including epithelial, endothelial and immune cells, fibroblasts and pericytes. Because we performed RNA-seq on the whole tissue, we cannot exclude that subtle yet significant differences in gene expression in specific subsets of cells were undetected.

We report that a considerable number of the genes with higher expression in the CP of progressive MS patients relative to control CP are involved in hypoxia, highlighting a novel aspect of the disease pathology in the CP. Genes such as *ADM* [10, 55], *SERPINE1* [35, 67], *HK2* [60], *STC2* [40] and *SNHG15* [73] are upregulated upon hypoxia. The expression of these genes is regulated by the transcription factors HIF-1ɑ and EPAS1 (also known as HIF-2ɑ) [18, 20, 35, 40, 44, 60, 67]. The regulatory function of HIF-1ɑ and EPAS1 occurs at the protein level, as their mRNA levels are not increased in hypoxia [52, 80] (Supplementary 1c and 2a). In addition, we show the involvement in neurological disease of the previously undescribed long non coding RNA *HIF1A-AS3* (ENSG00000258667). *HIF1A-AS3* is one of the three antisense genes of *HIF1A*. Another antisense, *HIF1A-AS2*, is involved in the regulation of HIF-1ɑ [12, 43]. *HIF1A-AS2* mRNA was not significantly higher in the CP of progressive MS patients relative to that of controls, suggesting that *HIF1A-AS3* may act as an upstream regulator of the hypoxia responses observed in our study. Altogether, our data indicate that the HIF1 pathway is altered in the CP of progressive MS patients. We speculate that the transcriptomic profile of the CP in progressive MS could be influenced by an exposure to hypoxic conditions. A hypoxic environment in progressive MS could originate from a reduction in blood supply at the CP stroma, or as a response to diffusible factors from the CSF compartment. Oxidative stress occurs in MS brains [25, 81], and CSF from MS patients can induce oxidative stress [78]. This suggests that oxidative factors can diffuse from the brain parenchyma into the CSF compartment, reaching the CP epithelium and leading to the respiratory enzyme deficiencies observed in MS patients [9]. Our results point to such an important pathological mechanism occurring at the CP in progressive MS patients.

One of the functions of the CP is the maintenance of immune homeostasis in the CNS. Although CNS inflammation is less evident in the progressive phases of MS, immune activation occurs in both the relapsing-remitting and progressive MS CP [65, 77]. However, we only detected a subtle alteration in the immunological profile. CXCL2 is a chemokine for granulocytes and, interestingly, neutrophilic LCN2 is upregulated at the onset of EAE [49]. Although no neutrophil markers were differentially expressed in our RNA-seq data, our recent immunohistochemical characterization of immune cell populations in the CP showed an accumulation of granulocytes in progressive MS patients relative to controls [65]. Future studies should explore the involvement of granulocytes in CP-mediated MS pathology, including whether their release of reactive oxygen species is related to the hypoxia-like responses observed. Previous transcriptomic studies of the CP from other neurological diseases detected alterations in immune-related pathways [34, 70]. Moreover, our recent study indicated that CD8+ T cells are more abundant in progressive MS CP than in control, which we could not detect in the present work. These discrepancies suggest that our bulk RNA-seq approach does not have enough resolution for detecting changes in relatively scarce cell populations, such as are granulocytes and T cells in the CP. Altogether, our dataset supports the view that strong inflammatory reactions are absent from the progressive MS CP.

Another major role of the CP is the formation of the BCSFB at the epithelial tight junctions. In contrast to previous studies [37], we did not identify clear alterations in the barrier properties of the CP in progressive MS patients. This is in line with other research that showed a lack of involvement of the junctional protein claudin-3 in EAE [13]. Thus, the structural BCSFB properties in progressive MS patients may remain intact and support CNS homeostasis.

The secretion of CSF is the main function of the CP. The CP has a strategic location to secrete signaling molecules that can reach the CNS periventricular areas, as well as more distant loci such as the cortex or spinal cord. To date, however, secretory alterations of the CP in neurological diseases have not been sufficiently investigated. Our transcriptome analysis identified three genes coding for secreted proteins with higher expression in the CP of progressive MS patients. At the protein level, PAI-1 was present in higher concentrations in CSF samples from MS patients with high disability relative to those with low disability. Our findings indicate that CP epithelial cells may produce ADM and PAI-1, in line with previous studies [71, 72, 76, 79]. Data from our lab and a publicly available dataset suggest that the origin of the CSF protein alterations is the CP and not the brain parenchyma.

PAI-1 (coded by *SERPINE1*) is a secreted proteinase inhibitor involved in the regulation of fibrinolysis. A genotype associated with low PAI-1 production [53] is a risk factor for MS [47, 84], illustrating its neuroprotective properties. Indeed, PAI-1 has an anti-apoptotic function in neurons [69] and it increases BBB tightness [14]. However, in a chronic relapsing EAE model, *SERPINE1* knockout mice showed less disease severity than the wild types [15]. Higher PAI-1 concentrations have been previously detected in the CSF of MS patients compared to that in controls [1]. Moreover, higher *SERPINE1* expression was detected in the CP of Alzheimer’s and Huntington’s disease and frontotemporal dementia patients relative to controls [70]. Our findings suggest that in the most severe cases of progressive MS the CP secretes higher levels of PAI-1 into the CSF, where it can travel into lesion-prone periventricular areas in an attempt to promote neuroprotection. Interestingly, higher levels of PAI-1 have been detected in acute MS lesions compared to control tissue [26]. ADM is a neuroprotective vasodilator [23, 24] that can promote oligodendrocyte differentiation [48] and tightening of the BBB [36]. ADM has a therapeutic potential based on its protective effects in EAE [56]. This neuroprotective mechanism does not seem specific for MS, but rather a general response to CNS damage, as ADM concentration is also elevated in the CSF after traumatic brain injury [62] and in ageing cortex [38]. More samples would be needed to determine if ADM is upregulated in the CSF of MS patients.

Previous studies have pointed to the neuroprotective role of the CP [6, 16, 17, 28]. In addition to *ADM* and *SERPINE1* (PAI-1), other genes related to neuroprotection presented higher expression in progressive MS CP relative to control CP. STC2 is a glycoprotein with neuroprotective effects in hippocampal degeneration [8]. Although the concentration of STC2 in the human CSF samples analyzed did not reflect the gene expression, this could be due to a restricted local expression of the peptide. Moreover, two metallothionein genes, *MT1A* and *MT1X*, presented higher expression in progressive MS CP than in control CP. Metallothioneins are antioxidant metal binding molecules recently explored as therapeutic agents [31]. The increased *MT1X* expression in progressive MS CP may be caused by hypoxia [68, 75], or by the higher copper levels present in the CSF of MS patients relative to healthy subjects [50]. Overall, we describe a neuroprotective signature in the CP of progressive MS patients which may reflect the role of this strategical tissue in restoring brain homeostasis in neurological disease.

Due to its homeostatic secretory activity, the CP is an interesting candidate as a therapeutic target. An attracting possibility would be to peripherally promote the local production of neuroprotective peptides, such as those identified here, at the CP and their secretion into the CSF. We observe a dysregulation of the HIF1 pathway in progressive MS CP, accompanied by a higher expression of several neuroprotective genes. Although hypoxic preconditioning can trigger neuroprotective responses [75], a pharmaceutical potentiation of these pathways bypassing the hypoxic exposure would reduce CNS damage. A recent study has described the cannabidiol quinone derivative drug VCE-004.8 as a novel therapeutic tool in MS, partly through the stabilization of HIF-1α and EPAS1 transcription factors and the activation of the HIF1 pathway [54]. Our findings suggest that the CP may be a key player in the protective functions of this promising drug, which is currently in phase I clinical trials.

In summary, we here provide comprehensive evidence for the involvement of the CP in progressive MS. Transcriptomic alterations related to hypoxic responses and the secretion of neuroprotective peptides illustrate the ability of the CP to monitor and respond to changes in the CNS environment. This study highlights the homeostatic capacities and disease alterations of the CP and suggests that manipulating its secretory properties may influence MS disease pathogenesis and promote neuroprotection.

## Supplementary information

**Additional file 1: Supplementary Fig. 1** Transcriptional profile of the human choroid plexus in progressive MS. **a** Validation by RT-qPCR of genes identified to be differentially expressed by RNA-seq. mRNA expression levels were normalised to the average of two stable reference genes, namely *GAPDH* and *18 s rRNA*. Each dot corresponds to an individual gene, coloured according to the RT-qPCR *p*-value. Differences were tested by two-tailed Welch t-test. **b** Expression in Fragments Per Kilobase Million (FPKM) of the EPAS1–202 isoform in progressive MS and control CP, as assessed by RNA-seq. **c** Normalised expression of the genes *EPAS1* and *HIF1A* in progressive MS and control CP, as assessed by RNA-seq **d** PCA plot of RNA-seq samples illustrates the high variability in the transcriptional profile. The percentage of data variation explained by the first two principal components (PC1 and PC2) is displayed. Each dot corresponds to an individual sample. PC1 explains most of the sample variation between control and progressive MS cases.

**Additional file 2: Supplementary Fig. 2** Hypoxia responses in human CP explants. **a***HIF1A* and *HIF1A-AS3* difference in relative gene expression between human postmortem CP paired samples from each donor (*n* = 6) incubated 24 h in hypoxia (1% O_2_) or normoxia (20% O_2_) analyzed using qPCR. Data are normalised to *18 s rRNA*. Results are displayed as the negative difference in ct values between hypoxia and normoxia (represented as -ΔΔct) and presented as median with confidence interval. Differences were tested by paired two-tailed Welch t-test.

**Additional file 3: Supplementary Fig. 3** Altered concentration of ADM and PAI-1 peptides in the CSF of MS patients. **a** Correlation plot of ADM protein concentration with age in CSF samples (*n* = 53). Smoothing line is calculated with linear regression, and shadow area represents the confidence interval around the smooth line. **b** Age of CSF donors with MS (*n* = 27) or control (*n* = 26), used for ADM protein assessment. Results are displayed as median and interquartile ranges (IQR). Differences were tested by Welch Two Sample t-test. **c** ADM protein concentration in the CSF of MS patients with high disability (high EDSS; *n* = 9) or low disability (low EDSS; *n* = 18), as measured by RIA. Results are displayed as median and interquartile ranges (IQR). Differences were tested by Wilcoxon rank sum test with continuity correction. **d** Age of CSF donors with high disability (high EDSS; *n* = 9) or low disability (low EDSS; n = 18), used for ADM protein assessment. Results are displayed as median and interquartile ranges (IQR). Differences were tested by Welch Two Sample t-test. **e** Correlation plot of PAI-1 protein concentration with age in CSF samples (*n* = 31). Smoothing line is calculated with linear regression, and shadow area represents the confidence interval around the smooth line. **f** Age of CSF donors with high disability (high EDSS; *n* = 9) or low disability (low EDSS; *n* = 7), used for PAI-1 protein assessment. Results are displayed as median and interquartile ranges (IQR). Differences were tested by Welch Two Sample t-test. **g** Correlation plot of STC2 protein concentration with age in CSF samples (*n* = 37). Smoothing line is calculated with linear regression, and shadow area represents the confidence interval around the smooth line. **h** STC2 protein concentration in the CSF of MS patients with high disability (high EDSS; n = 7) or low disability (low EDSS; n = 8), as measured by ELISA. Results are displayed as median and interquartile ranges (IQR). Differences were tested by Welch Two Sample t-test. **i** Age of CSF donors with high disability (high EDSS; n = 7) or low disability (low EDSS; n = 8), used for STC2 protein assessment. Results are displayed as median and interquartile ranges (IQR). Differences were tested by Welch Two Sample t-test.

**Additional file 4: Supplementary Fig. 4** Microarray gene expression data extracted from GEO dataset GSE38010. The expression values of the different samples from white matter from healthy controls (‘Control) or plaques from MS brains (‘Early’, ‘Active’, ‘Chronic’) are shown a) For ADM, one probe was available (202912_at) b) For SERPINE1 there were two probes available (202627_s_at and 202628_s_at) c) For STC2 there were two probes available (203438_at and 203439_s_at)

**Additional file 5: Supplementary Table 1.** Details of the human choroid plexus samples used for RNA-seq. F: female, M: male; PMD: Post-mortem delay; RIN: RNA integrity number; SP: Secondary Progressive; PP: Primary Progressive. The type of MS is noted in between brackets, as extracted from the clinical report, sometimes unclear

**Additional file 6: Supplementary Table 2.** Details of the human choroid plexus samples used for explants culture. F: female, M: male; PMD: Post-mortem delay.

**Additional file 7: Supplementary Table 3.** Details of the human CSF samples used. Patient information is provided, together with an indication of the experiment(s) for which the samples were used. NINDC: non-inflammatory disease controls; SC: symptomatic controls; INDC: inflammatory CNS disease.

**Additional file 8: Supplementary Table 4**. Details of the human brain samples used. Patient information and lesion type are provided. PMD: Post-mortem delay; CA: chronic active; CIA: chronic inactive.

**Additional file 9: Supplementary Table 5.** Primer sequences used in qPCR.

**Additional file 10: Supplementary Table 6.** Gene ontology enrichment analysis. Table displays the GO category ID, the name of the term, the number of differentially expressed (DE) genes in the category and the total number of genes in the category.

**Additional file 11: Supplementary Table 7.** Results from the gene expression analysis in CP explants in normoxia and hypoxia. Table displays the *p*-values of the difference in expression between the CP explants in normoxia or hypoxia, assessed by paired two-tailed Welch t-test

## Data Availability

The RNA-seq dataset is available in the GEO database under the accession number GSE137619.
