# Supplementary figures and images for: Altered secretory and neuroprotective function of the choroid plexus in progressive multiple sclerosis
Source: Acta Neuropathol Commun. 2020 Mar 19;8:35. doi: 10.1186/s40478-020-00903-y (PMC7083003; doi:10.1186/s40478-020-00903-y)

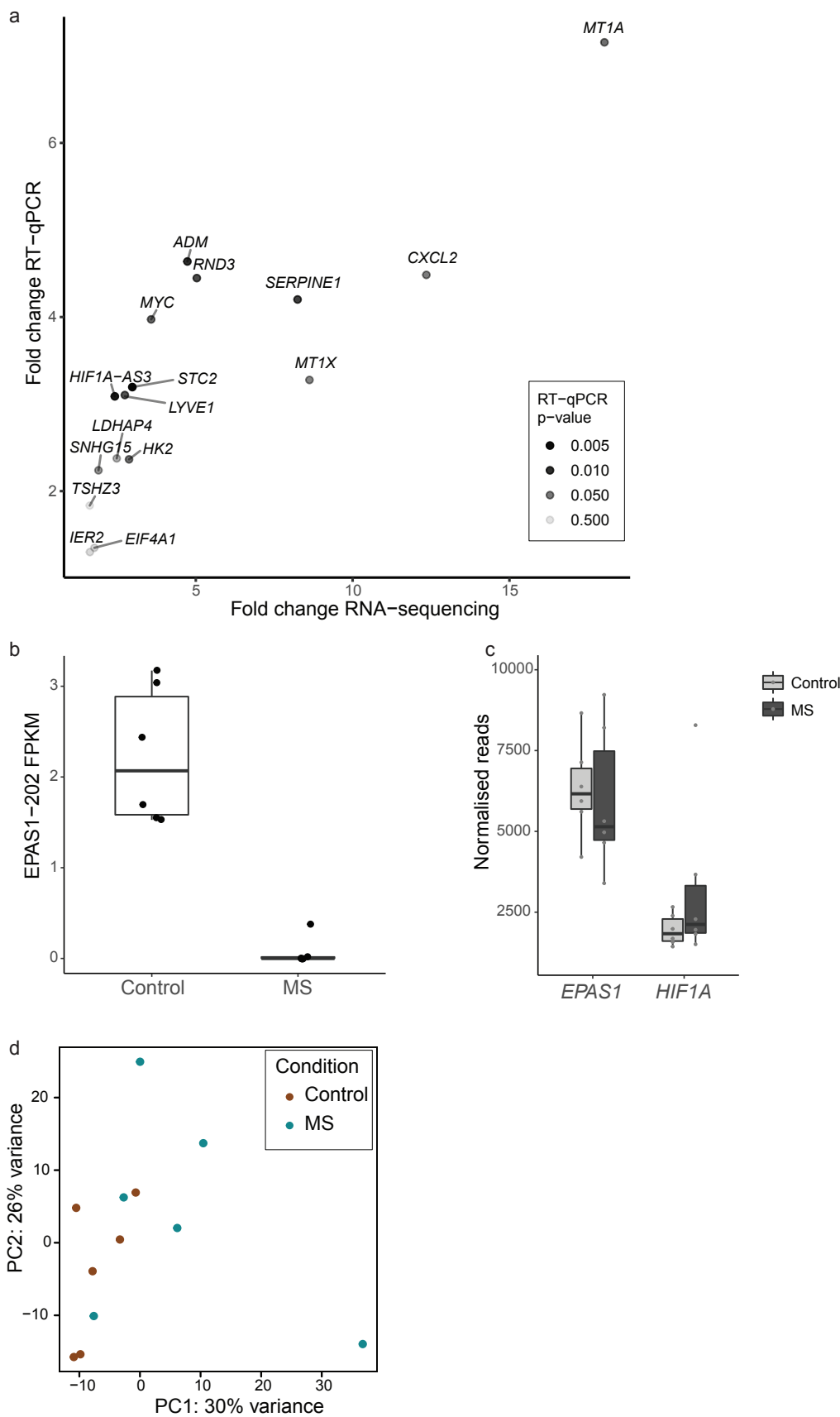

Supplement: Supplementary file 1 — Additional file 1: Supplementary Fig. 1 Transcriptional profile of the human choroid plexus in progressive MS. a Validation by RT-qPCR of genes identified to be differentially expressed by RNA-seq. mRNA expression levels were normalised to the average of two stable reference genes, namely GAPDH and 18 s rRNA. Each dot corresponds to an individual gene, coloured according to the RT-qPCR p-value. Differences were tested by two-tailed Welch t-test. b Expression in Fragments Per Kilobase Million (FPKM) of the EPAS1–202 isoform in progressive MS and control CP, as assessed by RNA-seq. c Normalised expression of the genes EPAS1 and HIF1A in progressive MS and control CP, as assessed by RNA-seq d PCA plot of RNA-seq samples illustrates the high variability in the transcriptional profile. The percentage of data variation explained by the first two principal components (PC1 and PC2) is displayed. Each dot corresponds to an individual sample. PC1 explains most of the sample variation between control and progressive MS cases. [file 40478_2020_903_MOESM1_ESM.pdf]

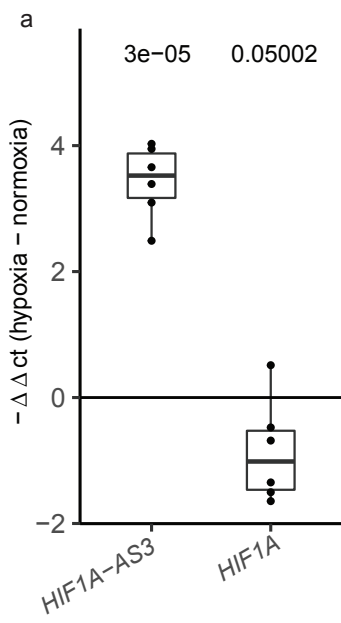

Supplement: Supplementary file 2 — Additional file 2: Supplementary Fig. 2 Hypoxia responses in human CP explants. aHIF1A and HIF1A-AS3 difference in relative gene expression between human postmortem CP paired samples from each donor (n = 6) incubated 24 h in hypoxia (1% O2) or normoxia (20% O2) analyzed using qPCR. Data are normalised to 18 s rRNA. Results are displayed as the negative difference in ct values between hypoxia and normoxia (represented as -ΔΔct) and presented as median with confidence interval. Differences were tested by paired two-tailed Welch t-test. [file 40478_2020_903_MOESM2_ESM.pdf]

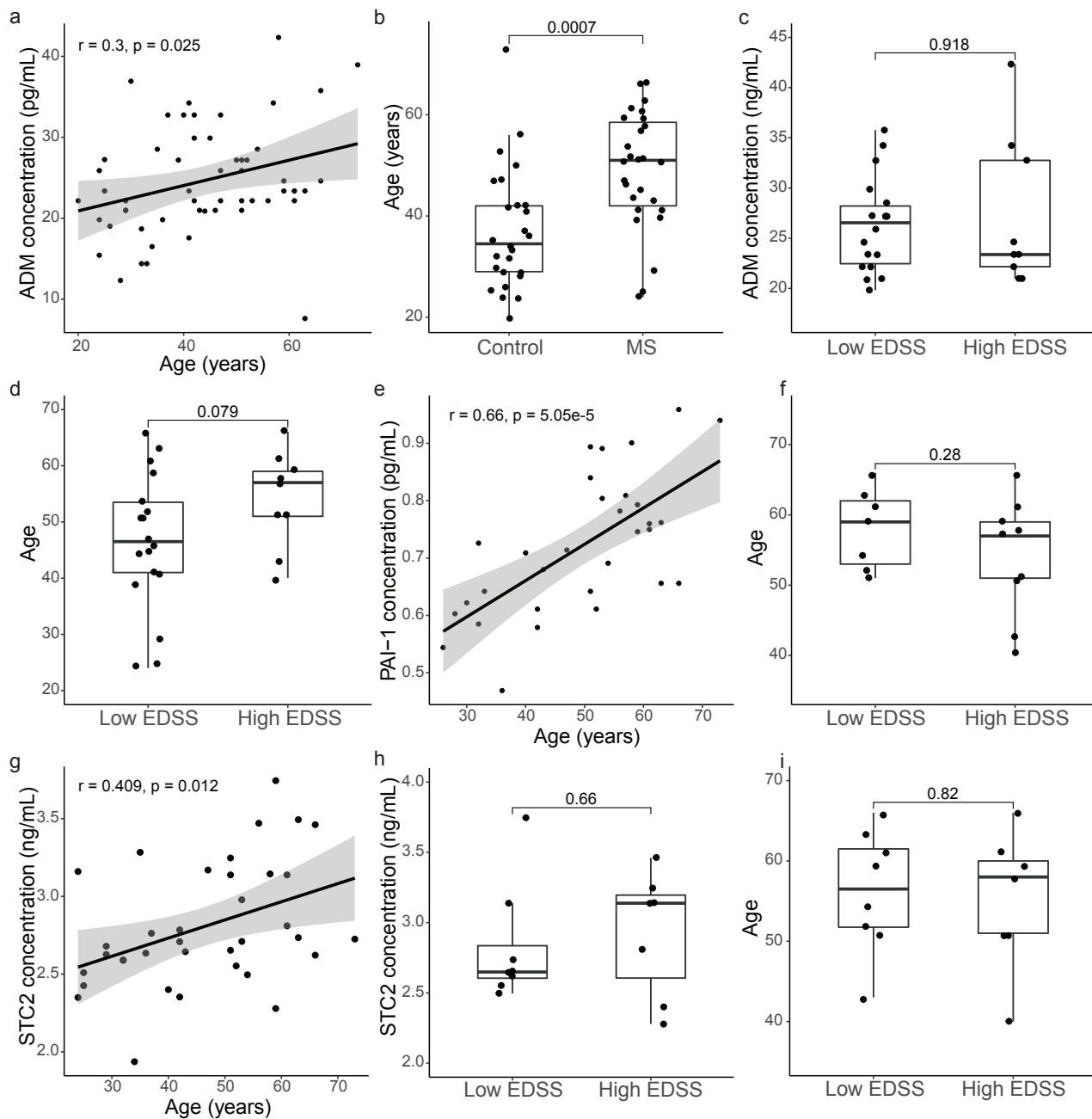

Supplement: Supplementary file 3 — Additional file 3: Supplementary Fig. 3 Altered concentration of ADM and PAI-1 peptides in the CSF of MS patients. a Correlation plot of ADM protein concentration with age in CSF samples (n = 53). Smoothing line is calculated with linear regression, and shadow area represents the confidence interval around the smooth line. b Age of CSF donors with MS (n = 27) or control (n = 26), used for ADM protein assessment. Results are displayed as median and interquartile ranges (IQR). Differences were tested by Welch Two Sample t-test. c ADM protein concentration in the CSF of MS patients with high disability (high EDSS; n = 9) or low disability (low EDSS; n = 18), as measured by RIA. Results are displayed as median and interquartile ranges (IQR). Differences were tested by Wilcoxon rank sum test with continuity correction. d Age of CSF donors with high disability (high EDSS; n = 9) or low disability (low EDSS; n = 18), used for ADM protein assessment. Results are displayed as median and interquartile ranges (IQR). Differences were tested by Welch Two Sample t-test. e Correlation plot of PAI-1 protein concentration with age in CSF samples (n = 31). Smoothing line is calculated with linear regression, and shadow area represents the confidence interval around the smooth line. f Age of CSF donors with high disability (high EDSS; n = 9) or low disability (low EDSS; n = 7), used for PAI-1 protein assessment. Results are displayed as median and interquartile ranges (IQR). Differences were tested by Welch Two Sample t-test. g Correlation plot of STC2 protein concentration with age in CSF samples (n = 37). Smoothing line is calculated with linear regression, and shadow area represents the confidence interval around the smooth line. h STC2 protein concentration in the CSF of MS patients with high disability (high EDSS; n = 7) or low disability (low EDSS; n = 8), as measured by ELISA. Results are displayed as median and interquartile ranges (IQR). Differences were tested by Welch Two S [file 40478_2020_903_MOESM3_ESM.pdf]

Supplementary 4

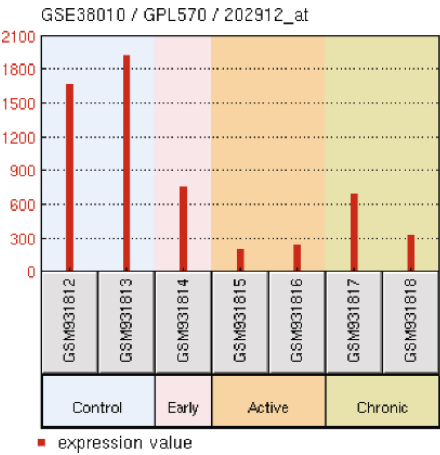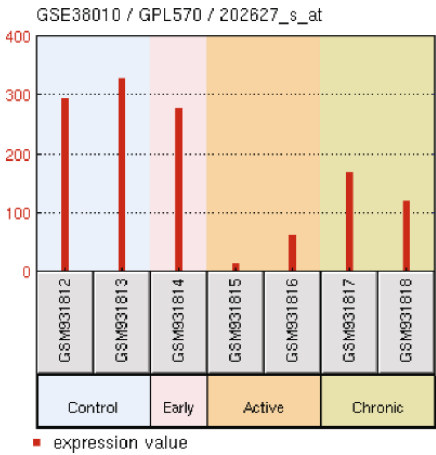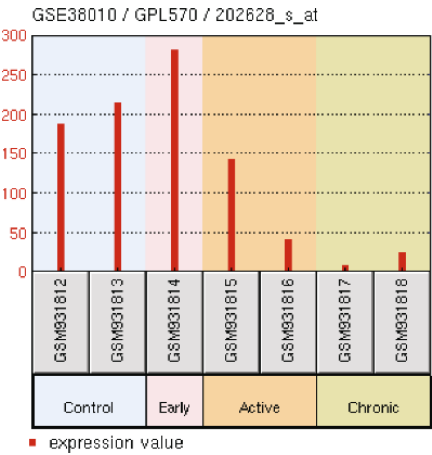

Supplement: Supplementary file 4 — Additional file 4: Supplementary Fig. 4 Microarray gene expression data extracted from GEO dataset GSE38010. The expression values of the different samples from white matter from healthy controls (‘Control) or plaques from MS brains (‘Early’, ‘Active’, ‘Chronic’) are shown a) For ADM, one probe was available (202912_at) b) For SERPINE1 there were two probes available (202627_s_at and 202628_s_at) c) For STC2 there were two probes available (203438_at and 203439_s_at) [file 40478_2020_903_MOESM4_ESM.pdf]
